# The association between maternal nutrition and lifestyle during pregnancy and 2-year-old offspring adiposity: analysis from the ROLO study

**DOI:** 10.1007/s10389-016-0740-9

**Published:** 2016-06-09

**Authors:** Mary K. Horan, Jean M. Donnelly, Ciara A. McGowan, Eileen R. Gibney, Fionnuala M. McAuliffe

**Affiliations:** 1 UCD Obstetrics and Gynaecology, School of Medicine and Medical Science, University College Dublin, National Maternity Hospital, Dublin 2, Ireland; 2 Science Centre – South, University College Dublin School Of Agriculture & Food Science, Belfield, Dublin 4 Ireland

**Keywords:** Foetal programming, Maternal nutrition, Childhood obesity, Glycaemic index

## Abstract

**Aim:**

To examine the association between maternal nutrition and lifestyle factors and offspring adiposity, using baseline and 2-year postpartum follow-up data from a randomised control trial of low glycaemic index diet.

**Subject and methods:**

Food diaries and lifestyle questionnaires were completed during pregnancy and infant feeding and maternal lifestyle questionnaires 2 years postpartum for 281 mother and infant pairs from the ROLO study. Maternal anthropometry was measured throughout pregnancy and infant and maternal anthropometry was measured 2 years postpartum.

**Results:**

Maternal 2 year postpartum body mass index (BMI) was positively associated with offspring BMI-for-age z-score (*B* = 0.105, *p* = 0.015). Trimester 2 saturated fat intake was positively associated with offspring subscapular:triceps skinfold ratio (*B* = 0.018, *p* = 0.001). Trimester 1 glycaemic index was also positively associated with offspring sum of subscapular and triceps skinfolds (*B* = 0.009, *p* = 0.029).

**Conclusions:**

Maternal BMI 2 years postpartum was positively associated with offspring BMI. Pregnancy saturated fat intake was positively and polyunsaturated fat negatively associated with offspring adiposity. While further research is necessary, pregnancy and the postpartum period may be early opportunities to combat childhood obesity.

**Electronic supplementary material:**

The online version of this article (doi:10.1007/s10389-016-0740-9) contains supplementary material, which is available to authorized users.

## Introduction

While the importance of the in utero environment throughout the different stages of pregnancy is well established for normal foetal growth and development (Wu et al. 2004), there is increasing interest in its role in foetal programming particularly in the area of maternal over-nutrition as rates of maternal overweight and obesity increase worldwide (Alexandra et al. 2011; Black et al. 2013; Fisher et al. 2013; Lau et al. 2011; Reynolds et al. 2010; Rogers 2003). Foetal programming of overweight and obesity appears to occur in large for gestational weight infants as well as small for gestational weight infants resulting in a U- or J-shaped curve of higher childhood and adulthood body mass index (BMI) at both extremes of high and low birthweight (Barker 2004; Curhan et al. 1996a, 1996b; Parsons et al. 2001; Rogers 2003; Sayer et al. 2004). Macrosomia results in greater risk of childhood (Blair et al. 2007; Oken and Gillman 2003; Yu et al. 2007) and adulthood (Serdula et al. 1993; Srinivasan et al. 1996) overweight and obesity as overweight and obesity track from infancy throughout life (Catalano and Ehrenberg 2006; Rogers 2003; Sayer et al. 2004).

To date, some studies have found that specific aspects of maternal diet are associated with offspring adiposity such as protein intake (Cuco et al. 2006; Maslova et al. 2014a, 2014b), saturated (Horan et al. 2014c; Murrin et al. 2013), trans (Cohen et al. 2011; Dirix et al. 2009) and polyunsaturated (De Vries et al. 2014; Donahue et al. 2011; Hauner et al. 2012; Moon et al. 2012) fatty acid intake and dietary glycaemic index (GI; Horan et al. 2014c; Moses et al. 2006; Okubo et al. 2014; Walsh et al. 2012b). However, study results are often conflicting (Brion et al. 2010; Moses et al. 2014; Walsh et al. 2012b).

In addition, there has been little research into the effect of low GI healthy eating interventions in pregnancy on maternal weight postpartum in euglycaemic women. Interventions in gestational diabetes mellitus (GDM) have shown little success after pregnancy (Fehler et al. 2007; Louie et al. 2015; Stage et al. 2004). Mothers from the ROLO (Randomised cOntrol trial of LOw GI diet versus no dietary intervention to prevent recurrence of foetal macrosomia) study had reduced weight gain from early pregnancy and greater reported dietary health behaviours, e.g. food label reading, at 3 months postpartum but there was no significant difference in weight or BMI between the control and intervention groups at this time-point (Horan et al. 2014b).

The aim of this study was to determine which, if any, maternal characteristics and pregnancy dietary factors were associated with infant adiposity at 2 years of age in a cohort of the ROLO study (Walsh et al. 2012b) and to examine the effect of a low GI dietary intervention in pregnancy on maternal BMI 2 years postpartum.

## Methods

Two hundred and eighty one mother and infant pairs from the ROLO study were included in this analysis. These mother and infant pairs were participants of the original ROLO study who agreed to return for follow-up at 2 years postpartum. The original ROLO study consisted of 800 secundigravida women who had previously given birth to a macrosomic baby (>4 kg) and were, therefore, at high risk of delivering another macrosomic infant (Mahony et al. 2006). A power study performed when designing the ROLO study found that 360 women in each group (control and intervention) would be needed to detect a 0.25 standard deviation difference in the primary outcome; birth weight, with 90 % power. Therefore, a sample size of 800 was considered sufficient to allow for drop-outs. The women were randomised to receive low GI dietary advice in early pregnancy or usual antenatal care, which did not include dietary advice. Detailed methodology and results of the ROLO study have previously been published (Walsh et al. 2010, 2012a). In brief, women were randomised using computer generated allocations in a ratio of 1:1 stored in sealed opaque envelopes. Once randomised, it was not possible to blind participants or researchers to study group affiliation. Women from the intervention group attended 1–2 hour dietitian-led low GI dietary advice sessions at week 14 of pregnancy in groups of 2–6 participants. Dietary education was designed to meet Irish nutritional recommendations for pregnant women (DOHC 2005). Women were not given specific information on their energy requirements or advice on gestational weight gain, however, they were advised not to ‘eat for two’ and to consume an additional 200–300 kcal per day in the last trimester of pregnancy. Women were educated on the definition of GI and the rationale for its use during pregnancy and written information was given about GI along with a list of foods with high and low GI values. Women were encouraged to choose low GI instead of high GI foods. A list of low GI recipes was also provided and women were emailed additional low GI recipes during their pregnancy. Demographic, well-being and lifestyle questionnaires were returned by 28 weeks gestation and 3-day food diaries were completed during each trimester of pregnancy. Maternal weight and height were recorded in early pregnancy and maternal weight was also measured throughout gestation. Offspring birthweight, length, circumferences and skinfold thicknesses were measured at birth. As mentioned, the primary outcome was birthweight, which was significantly lower in the intervention group. The secondary outcomes of reduced maternal gestational weight gain and insulin intolerance were also achieved.

### Inclusion and exclusion criteria

Participants were secundigravida women who had previously given birth to a macrosomic infant (>4 kg). They were required to have sufficient literacy and English language fluency to understand the intervention and complete questionnaires. The women had healthy, singleton pregnancies with no intrauterine growth abnormalities. All participants were invited to return for a follow-up appointment with their infant 2 years postpartum and infants were eligible for inclusion until they reached 2 years 6 months old.

### Maternal demographics, lifestyle and infant feeding practices

Of the 800 participants of the ROLO study, 281 returned at 2 years postpartum. Questions on lifestyle habits were taken from the SLAN (Survey of Lifestyle, Attitudes and Nutrition in Ireland) (Harrington et al. 2008) questionnaire including questions on physical activity, smoking, education and food label reading.

### Maternal and infant anthropometry

Maternal weight, height and mid-upper arm circumference were measured at the first antenatal consultation and BMI calculated. Maternal weight was measured at each subsequent consultation and gestational weight gain calculated. Maternal weight and mid-upper arm circumference were also measured 2 years postpartum and BMI calculated.

Infant weight, length, mid-upper arm, abdominal, hip and thigh circumference, and biceps, triceps, subscapular and thigh skinfold thickness were measured at 2 years of age. World Health Organisation (WHO) growth standards were used to convert these measurements to z-scores which adjusts for infant age at exam and gender and report standard deviations away from the median (Horan et al. 2014a; WHO 2006). Waist:hip, waist:length and subscapular:triceps skinfold ratios as well as sum of triceps and subscapular skinfold thicknesses and sum of all skinfold thicknesses were calculated as markers of infant adiposity.

### Maternal dietary intakes during pregnancy

Three-day food diaries were completed during each trimester of pregnancy and used to determine macronutrient intake as well as the GI and load of the women’s diet. Macronutrients were expressed as a percentage of total energy. Dietary intake in each trimester of pregnancy was examined separately. Underreporting was estimated using Goldberg ratios, i.e. the ratio of energy intake to estimated basal metabolic rate (Goldberg et al. 1991). Basal metabolic rate was calculated using Schofield equations and a Goldberg ratio of ≤0.9 was used to identify underreporters (Goldberg et al. 1991; McGowan and McAuliffe 2012; Schofield 1984). Full methodology for entry and analysis of the dietary intake of participants has previously been published (McGowan et al. 2013).

### Statistical analysis

Statistical analysis was completed using SPSS (Statistical Package for the Social Sciences) software version 20.0. Control and intervention groups were combined for analysis but study group was controlled for in all final models. Correlations, independent sample t-tests, chi-squared tests and analysis of variance (ANOVA) was used as appropriate to identify maternal characteristics and macronutrient intakes associated with infant adiposity at 2 years of age (*p* < 0.05). Variables examined included macronutrient intake for each trimester, parental height, weight and BMI during pregnancy and maternal weight and BMI at 2 years months postpartum, gestational weight gain, reported maternal physical activity and smoking during pregnancy. Maternal physical activity, smoking and alcohol intake was also reported at 2 years postpartum. Data on maternal ethnicity, maternal age at delivery, marital/partner status and GI and load was also recorded during pregnancy. Variables found to be significantly associated with infant anthropometry at 2 years of age were further analysed using simple linear regression then input into final multiple regression models using a forced enter and backwards stepwise approach. This resulted in any non-significant variables being discarded from the model in a stepwise manner. Variables known to affect infant adiposity (birthweight, maternal education level as a marker of socioeconomic status, age in weeks at 2-year follow-up exam, infant gender and breastfeeding duration) were controlled for using forced enter multiple regression in these models. Models predictive of infant anthropometric measurements for which z-scores were available were not adjusted for infant age and gender as these factors are taken into account when converting to z-scores. Membership of the control or intervention group and maternal dietary underreporting (Goldberg ratio ≤0.9) were also included by forced enter multiple regression in these models and analysis was carried out excluding underreporters. Multiple linear regression resulted in a best and final model and models that were statistically significant overall (*p* < 0.05) were chosen as those which best predicted neonatal anthropometric measurements. Maternal weight and BMI was compared between the control and intervention groups at 2 years postpartum using independent sample t-tests.

## Results

### Maternal demographics, lifestyle and infant feeding practices

Maternal demographic, anthropometric and lifestyle characteristics at baseline and 2-year follow-up are displayed in Table 1. Women who returned at 2 years postpartum were aged 32.96 ± 3.92 years at delivery, 92.3 % of them were of white Irish ethnicity while 6.8 % were of white non-Irish and 0.3 % were each of Chinese, Indian and Filipino/South East Asian ethnicity. Fifty-nine percent had completed third level education, while 22 % had completed some third level education, 15.3 % had completed second level education and 3.7 % had completed some second level education. Ninety-seven point nine percent of women reported they were non-smokers in early pregnancy, while 2.1 % reported smoking. At 2 years postpartum 86.5 % reported they were non-smokers, while 13.5 % reported smoking. Maternal BMI in early pregnancy was 26.22 ± 4.54 kg/m^2^, while at 2 years postpartum maternal BMI was 25.95 ± 4.50 kg/m^2^. There was no difference between the control and intervention group at either time-point in maternal BMI (*p* = 0.524 and *p* = 0.311 respectively). While maternal gestational weight gain was lower in the intervention group in the original ROLO study, gestational weight gain in this cohort was 13.59 ± 4.61 and did not differ significantly between the control and intervention groups (*p* = 0.317).

**Table 1:** Baseline and 2-year follow-up maternal demographic and lifestyle characteristics

	Total mean	SD	*p*-value
Mother age at delivery (years)	32.96	3.92	0.325
Baseline strenuous activity (No. of 20-min intervals/week)	2.00	1.02	0.288
Baseline moderate activity (No. of 20-min intervals/week)	3.13	1.99	0.020
Baseline mild activity (No. of 20-min intervals/week)	4.01	2.37	0.218
Mother height (cm)	165.44	14.17	0.893
Mother weight (kg)	72.60	13.33	0.533
Mother mid upper arm circumference at baseline (cm)	29.27	3.31	0.297
Gestational weight gain (kg)	13.59	4.61	0.317
Mother BMI baseline (kg/m^2^)	26.22	4.54	0.524
Mother weight at 2 years (kg)	71.71	13.43	0.307
Mother mid-upper arm circumference at 2 years (cm)	29.11	3.60	0.769
Mother BMI at 2 years (kg/m^2^)	25.95	4.50	0.311
Duration of breastfeeding (weeks)	13.04	23.23	0.082
Age given drinks other than breastmilk (weeks)	12.59	16.66	0.937
2-year strenuous activity (No. of 20-min intervals/week)	0.91	1.46	0.106
2-year moderate activity (No. of 20 min intervals/week)	2.12	2.44	0.012
2-year mild activity (No. of 20 min intervals/week)	2.26	2.88	0.752

Comparison of control and intervention groups carried out using independent sample t-tests

*BMI* body mass index

With regard to infant feeding practices; 64.9 % of women reported breastfeeding their second child while 35.1 % did not breastfeed. The mean duration of breastfeeding was 13.04 ± 23.23 weeks. The age infants were first given drinks other than breast milk (including formula milk) was 12.59 ± 16.66 weeks, while solids were reportedly first introduced at 19.56 ± 5.08 weeks. There was no significant difference between the control and intervention groups in infant feeding practices.

### Loss to follow-up

A comparison of baseline characteristics of participants and those lost to follow-up is displayed in electronic supplementary material (ESM1). The only significant difference observed between the groups was a greater reported frequency of 20-min intervals of moderate physical activity per week in those lost to follow-up (3.57 ± 2.44 vs 3.00 ± 1.85 intervals, *p* = 0.020).

### Infant anthropometry

Infant anthropometric characteristics are displayed in Table 2. Infant adiposity, estimated using ratios and sums of infant anthropometry measurements, did not differ between the control and intervention groups except for hip circumference which was lower in the control group in this cohort which only contained participants who had both completed food diaries during pregnancy and returned at 2-years postpartum (49.02 ± 3.55 vs 49.96 ± 3.22). Classification of infants according to WHO BMI-for-age cut-offs (WHO 2006) resulted in 4 (1.4 %) being classified as “wasted”, 204 (72.1 %) as “normal”, 51 (18 %) as “at risk of overweight”, 22 (7.8 %) as “overweight” and 2 (0.7 %) as “obese”.

**Table 2:** Anthropometric characteristics of offspring at birth and at 2 years of age

	*n *(intervention)	*n* (total)	Mean	SD
Birth length (cm)	116	234	52.94	2.21
Birth head circumference (cm)	67	132	35.83	1.20
Birth abdominal circumference (cm)	69	137	33.56	1.92
Birth thigh circumference (cm)	69	137	16.21	1.70
Birth chest circumference (cm)	69	137	35.58	2.28
Birth hip circumference (cm)	68	136	33.76	2.32
Birth mid-upper arm straight (cm)	69	137	12.53	1.15
Birth subscapular skinfold thickness (mm)	54	114	6.90	1.53
Birth triceps skinfold thickness (mm)	54	114	6.99	1.56
Birth biceps skinfold thickness (mm)	54	114	6.73	1.49
Birth thigh skinfold thickness (mm)	54	114	7.88	1.73
Birth weight (kg)	133	280	4.06	4.59
Birth sum of skinfold thicknesses (mm)	54	114	28.50	5.23
Birth waist:hip ratio	68	136	1.00	0.06
Birth sum of triceps and subscapular skinfold thickness (mm)	54	114	13.89	2.76
Birth subscapular:triceps skinfold thickness ratio	54	114	1.01	0.20
Birth waist circumference:length ratio	60	118	0.63	0.04
2 year length-for-age z-score	134	281	0.98	5.95
2-year head circumference-for-age z-score	130	276	1.37	1.37
2-year abdominal circumference (cm)	132	278	50.40	4.22
2-year thigh circumference (cm)	132	279	27.43	2.69
2-year chest circumference (cm)	127	271	50.59	2.91
2-year hip circumference (cm)	113	234	49.47	3.42
2-year mid-upper arm circumference-for-age z-score	132	279	1.13	1.12
2-year subscapular skinfold thickness-for-age z-score	112	219	0.73	1.12
2-year triceps skinfold thickness-for-age z-score	109	218	1.15	1.15
2-year biceps skinfold thickness (mm)	112	223	7.80	2.23
2-year thigh skinfold thickness (mm)	112	220	15.67	5.76
2-year weight-for-age z-score	134	281	0.69	1.01
2-year weight-for-length z-score	134	280	0.79	5.59
2-year BMI-for-age z-score	134	281	0.42	1.10
2-year waist circumference:length ratio	132	278	0.56	0.05
2-year waist:hip circumference ratio	113	234	1.02	0.08
2-year sum of skinfold thicknesses (mm)	112	216	40.82	8.14
2-year sum of triceps and subscapular skinfold thicknesses (mm)	112	218	17.46	3.40
2-year subscapular:triceps skinfold thickness ratio	112	218	0.74	0.21

*BMI* body mass index

### Associations between maternal body composition and lifestyle and offspring adiposity at 2 years of age

Unadjusted associations between maternal body composition and offspring adiposity at 2 years of age are displayed in ESM2 while adjusted associations are displayed in Table 3. Maternal BMI at 2 years postpartum was positively associated with BMI-for-age z-score (*B* = 0.105, *p* = 0.015). Maternal height was negatively associated with 2-year-old sum of all skinfold thicknesses, a measure of overall adiposity, (*B* = −0.371, *p* = 0.003) and with 2-year-old thigh skinfold thickness (*B* = −0.240, *p* = 0.004). In terms of lifestyle; 2-year-old waist:length ratio, a measure of central adiposity, was positively associated with maternal reported minutes sitting per weekday during early pregnancy (*B* = 4.71e-05, *p* = 0.019).

**Table 3:** Association of maternal and characteristics and maternal diet with offspring adiposity at 2 years of age- Multiple linear regression excluding underreporters*

	*B*	SEB	*p*	*R* ^2^adj	*F*	*P*
Weight-for-age z-score						
Mother MUA circumference baseline (cm)	0.065	0.035	0.065			
Trimester 3 saturated fat (%TE)	0.048	0.024	0.046	0.205	5.316	<0.001
2-year mother weight (kg)	0.030	0.014	0.035			
2-year mother BMI (kg/m^2^)	−0.111	0.042	0.009			
Age given drinks other than breast milk (weeks)	−0.010	0.005	0.057			
BMI-for-age z-score						
Trimester 1 GI	−0.045	0.018	0.014			
2-year mother weight (kg)	−0.025	0.015	0.087	0.081	3.340	0.002
2-year mother BMI (kg/m^2^)	0.105	0.043	0.015			
MUA circumference-for-age z-score						
Mother baseline smoker (yes/no)	2.603	0.881	0.004	0.078	3.277	0.008
Waist:length ratio						
Baseline maternal minutes sitting per weekday	4.71E-05	0.000	0.019			
Mother height (cm)	0.000	0.000	0.054	0.142	3.807	<0.001
Trimester 2 Polyunsaturated fat (%TE)	−0.005	0.002	0.017			
Trimester 3 Polyunsaturated fat (%TE)	−0.004	0.002	0.070			
Sum of all skinfold thicknesses						
Mother height (cm)	−0.371	0.122	0.003	0.070	2.365	0.020
Trimester 1 GI	−0.405	0.176	0.023			
Subscapular:triceps skinfold thickness ratio						
Trimester 1 GI	0.009	0.004	0.029	0.088	2.782	0.007
Trimester 2 Saturated Fat (%TE)	0.018	0.005	0.001			

Multiple linear regression analysis carried out. Adjusted for maternal education level, infant age in weeks at 2-year follow-up exam, infant gender, breastfeeding duration and study group. Maternal dietary underreporters removed (*Goldberg ratio ≤ 0.9). Maternal dietary intake derived from food diaries in each trimester of pregnancy

*TE* total energy, *GI* glycaemic index, *BMI* body mass index, *MUA* mid upper arm

### Associations between maternal macronutrient intake and GI during pregnancy and offspring adiposity at 2 years of age

Unadjusted associations between maternal dietary intake and offspring adiposity at 2 years of age are displayed in ESM2, while adjusted associations are displayed in Table 3. Maternal dietary intake during pregnancy is described in ESM3. A small number of maternal dietary factors were associated with several measures of offspring weight and adiposity at 2 years of age. Maternal trimester 2 polyunsaturated fat intake was negatively associated with 2-year-old waist:length ratio (*B* = −0.005, *p* = 0.017). Trimester 1 GI was negatively associated with 2-year old BMI-for-age z-score (*B* = −0.045, *p* = 0.014), hip circumference (*B* = −0.180, *p* = 0.026) and sum of all skinfold thicknesses (*B* = −0.405, *p* = 0.023).

## Discussion

The main findings of this follow-up study were that, whilst maternal BMI at 2 years postpartum was positively associated with 2-year-old BMI-for-age z-score, maternal height was negatively associated with 2-year-old sum of all skinfold thicknesses, a measure of overall adiposity. In terms of associations between maternal dietary factors and offspring adiposity at 2 years of age; trimester 3 saturated fat intake was positively associated with 2-year-old weight-for-age z-score. Conversely, maternal trimester 2 polyunsaturated fat intake was negatively associated with 2-year-old waist:length ratio, a measure of central adiposity. Maternal trimester 1 GI was negatively associated with 2-year-old BMI-for-age z-score, and sum of all skinfold thicknesses.

Our finding that infant BMI-for-age z-score at 2 years of age was positively associated with maternal BMI at 2 years postpartum was independent of maternal BMI in early pregnancy and of gestational weight gain, neither of which were significantly associated. This positive association between maternal and offspring adiposity has been observed in several studies (Gibson et al. 2007; Magarey et al. 2003; McDonald et al. 2009; Strauss and Knight 1999). Evidence shows that maternal, and indeed paternal, eating habits, lifestyle and nutritional knowledge influence that of family members in a variety of ways including behaviour modelling (Matheson et al. 2006; Turer et al. 2013), infant feeding practices (Clark et al. 2007; Li et al. 2003; Perrine et al. 2012) and gatekeeping of food-provision into the house, cooking methods and portion sizes served (Nguyen et al. 1996; Waxman and Stunkard 1980; Wild et al. 1994). Obese mothers are thought to exert less control over their children’s eating habits and structure at mealtimes than normal weight mothers (Baughcum et al. 2001; Wardle et al. 2002). Genetic factors are also likely to account in part for some of the positive associations between maternal and child overweight and obesity observed in the literature (Bouchard 2009). However, maternal overweight and obesity in the years post-partum has also been found to contribute to the positive correlation between maternal and offspring overweight (Jääskeläinen et al. 2011).

Maternal height was negatively associated with 2-year-old sum of all skinfold thicknesses, a measure of overall adiposity. Parental height is well known to affect offspring height with mid-parental height used to predict final offspring height (Aulchenko et al. 2009; Tanner et al. 1970). Parental height is correlated with socioeconomic status (Cardoso and Caninas 2010; Cavelaars et al. 2000; Subramanian et al. 2011) and higher rates of overweight and obesity in lower socioeconomic groups have been attributed in part to the lower energy requirements of individuals with shorter stature (Cecil et al. 2005; Heineck 2006; Lobstein and Frelut 2003). However, socioeconomic status is becoming less intrinsically linked to height as living conditions, food security and average height improve across the social strata and rather, parental height alone may now be the main predictor acting only as an artefact of ancestral socioeconomic status (Galobardes et al. 2012). Infant length-for-age z-score was not associated with maternal height in this cohort. This effect may be mediated by maternal higher socioeconomic status being associated with improved dietary and lifestyle habits postpartum (Gibbs and Forste 2014; Hupkens et al. 1998; Sallis et al. 1996; Shrewsbury and Wardle 2008; Vereecken et al. 2004; Wang 2001); however, these factors were not comprehensively examined by this study.

Maternal trimester 1 GI was negatively associated with 2-year-old BMI-for-age z-score and sum of all skinfolds. Several studies have found that maternal GI in pregnancy is positively associated with macrosomia (Clapp 1997; Moses et al. 2006; Scholl et al. 2004) and offspring adiposity (Donnelly et al. 2014; Okubo et al. 2014), while others have found no effect (Moses et al. 2007; Walsh et al. 2012a). A recent study by Okubo et al. (2014) found that maternal GI in early pregnancy was positively associated with offspring fat mass at 4 and at 6 years of age but not at birth. Observational studies focusing on sugars and carbohydrate intakes rather than GI have also found associations with offspring adiposity; Murrin et al. (2013) found that maternal sugar intake in early pregnancy was positively associated with offspring adiposity at 5 years of age while studies by Pereira-de-Silva et al. ( 2014) and Moore et al. (2004) found that maternal carbohydrate intake during pregnancy was positively associated with neonatal adiposity. Therefore, the negative relationship observed in this cohort was counterintuitive. However, when further analysis was carried out and the control and intervention groups were examined separately it was found that this negative association was only present in the control group. Therefore, it might be speculated that this relationship may have been attenuated by the low GI intervention in later pregnancy. Of course, the nature of this retrospective analysis precludes determination of causality and further studies would be required to explore any association further.

Maternal trimester 3 saturated-fat intake was positively associated with 2-year-old weight-for-age z-score. A study by Murrin et al. ( 2013) found that maternal trimester 1 saturated fat intake was positively associated with offspring overweight and obesity at 5 years of age while a study by Ladino et al. (2014) also found that maternal total fat intake was positively associated with offspring adiposity up to 18 months. Previously, we also observed a similar positive association at birth between neonate central adiposity and maternal trimester 3 saturated-fat intake (Horan et al. 2014c), while, at 6 months of age, central adiposity was found to be positively associated with trimester 2 saturated-fat intake (Horan et al. 2016).

Finally, there was no significant difference observed in maternal BMI between the control and intervention groups at 2 years postpartum. There is little data available on the effect of low GI or healthy eating diet in pregnancy on maternal weight postpartum with the exception of women with GDM. Pregnancy interventions in GDM have been found to have little impact on maternal dietary habits or weight postpartum despite knowledge of the mothers that GDM results in greater risk of later type II diabetes (Evans et al. 2010; Fehler et al. 2007; Louie et al. 2015; Stage et al. 2004). However, it should be noted that, unlike in the original ROLO study, there was no significant reduction in gestational weight gain in the intervention group of this cohort, who returned for follow-up at 2 years postpartum and had completed food diaries during pregnancy despite significant reduction in GI observed post-intervention.

This study had some limitations; an infant food frequency questionnaire would have allowed us to control for the dietary intake of the 2 year olds. However, we did have information on infant feeding and weaning practices and controlled for duration of breastfeeding in all final models. Another limitation was that data from this cohort may not be generalizable to other populations as this was a relatively well-educated group with a high incidence of macrosomia and concomitant risk of offspring obesity. This study had a high rate of loss to follow-up due to a delay in commencing 2-year old follow-up data collection as the study was not originally designed to be continued beyond 6 months postpartum. This, unfortunately, resulted in this study being inadequately powered to examine the effect of the intervention on offspring adiposity. However, loss to follow-up was largely logistic rather than to do with reluctance to return, and this study still involved a large cohort particularly suitable for examination of the association of maternal diet and lifestyle during pregnancy and offspring adiposity.

## Conclusion

Maternal BMI 2 years postpartum was positively associated with offspring adiposity at 2 years of age while maternal height was negatively associated. The quality of maternal dietary fat was a factor as saturated fat intake during pregnancy was positively associated with offspring adiposity. Finally, a low GI pregnancy intervention had no effect on maternal BMI 2 years postpartum. While further research is necessary, both pregnancy and the postpartum period may be early opportunities to combat childhood overweight and obesity.

## Electronic supplementary material

Below is the link to the electronic supplementary material.

ESM1 (DOCX 15 kb)

ESM2 (DOCX 20 kb)

ESM3 (DOCX 18 kb)
