# Supplementary material for: The association between maternal nutrition and lifestyle during pregnancy and 2-year-old offspring adiposity: analysis from the ROLO study
Source: Z Gesundh Wiss. 2016 Jun 9;24(5):427–36. doi: 10.1007/s10389-016-0740-9 (PMC5025498; doi:10.1007/s10389-016-0740-9)
Supplement: Supplementary file 1 — (DOCX 15 kb) [file 10389_2016_740_MOESM1_ESM.docx]

**Online Resource 1** A comparison of background characteristics of participants at 2 years postpartum and those lost to follow-up

|  | Participants | | | | Lost to Follow-up | | | | p-value |
| --- | --- | --- | --- | --- | --- | --- | --- | --- | --- |
|  | n | Mean | | SD | n | Mean | | SD |  |
| Mother age at delivery (yrs) | 253 | 33.18 | | 3.82 | 237 | 32.61 | | 4.03 | 0.105 |
| Mother BMI baseline kg/m^2^ | 280 | 26.03 | | 4.36 | 271 | 26.54 | | 4.27 | 0.169 |
| Gestational weight gain (kg) | 144 | 13.50 | | 4.53 | 130 | 13.36 | | 4.65 | 0.796 |
| Mild physical activity (number of 20 min intervals/week) | 215 | 4.04 | | 2.42 | 210 | 4.02 | | 2.58 | 0.925 |
| Moderate physical activity (number of 20 min intervals/week) | 163 | 3.00 | | 1.85 | 151 | 3.57 | | 2.44 | 0.020 |
| Strenuous physical activity (number of 20 min intervals/week) | 26 | 2.08 | | 1.02 | 27 | 1.78 | | 0.97 | 0.279 |
| Trimester 2 energy intake (MJ) | 282 | 7.87 | | 1.82 | 272 | 7.90 | | 2.10 | 0.880 |
| Trimester 3 energy intake (MJ) | 278 | 8.02 | | 1.80 | 259 | 7.86 | | 2.01 | 0.324 |
| Trimester 2 GI | 266 | 56.96 | | 3.69 | 254 | 57.23 | | 4.19 | 0.432 |
| Trimester 3 GI | 266 | 56.69 | | 3.83 | 254 | 57.29 | | 4.05 | 0.084 |
| Birthweight centile | 265 | 72.94 | | 24.62 | 250 | 72.83 | | 23.71 | 0.959 |
|  | n | | % | | n | | % | |  |
| Achieved 3^rd^ Level Education | 159 | | 58.89 | | 137 | | 52.49 | | 0.162 |
| Smoker early pregnancy | 6 | | 2.14 | | 13 | | 4.80 | | 0.104 |
| Member of intervention group | 135 | | 47.87 | | 112 | | 41.48 | | 0.146 |

Only participants who had completed food diaries during pregnancy were included in this analysis

BMI Body Mass Index
