# Supplementary material for: The association between maternal nutrition and lifestyle during pregnancy and 2-year-old offspring adiposity: analysis from the ROLO study
Source: Z Gesundh Wiss. 2016 Jun 9;24(5):427–36. doi: 10.1007/s10389-016-0740-9 (PMC5025498; doi:10.1007/s10389-016-0740-9)
Supplement: Supplementary file 2 — (DOCX 20 kb) [file 10389_2016_740_MOESM2_ESM.docx]

**Online Resource 2.** Associations between maternal nutrition and lifestyle factors in pregnancy and offspring anthropometry at 2 years of age- unadjusted analysis

|  | **B** | **SEB** | **p** | **R2adj** |
| --- | --- | --- | --- | --- |
| **Weight-for-Age z-score** |  |  |  |  |
| Mother baseline smoker (y/n) | 0.899 | 0.376 | 0.017 | 0.014 |
| Mother height (cm) | 0.009 | 0.004 | 0.019 | 0.013 |
| Mother weight baseline (kg) | 0.014 | 0.004 | 0.000 | 0.034 |
| Mother MUAC baseline (cm) | 0.036 | 0.017 | 0.034 | 0.011 |
| Trimester 2 GI | -0.043 | 0.017 | 0.010 | 0.021 |
| Macrosomia (y/n) | -0.469 | 0.107 | 0.000 | 0.053 |
| Trimester 3 saturated fat (%TE) | 0.041 | 0.020 | 0.042 | 0.011 |
| Trimester 3 polyunsaturated fat (%TE) | -0.076 | 0.033 | 0.023 | 0.015 |
| 2 year mother weight (kg) | 0.016 | 0.004 | 0.000 | 0.049 |
| 2 year mother BMI (kg/m^2^) | 266.596 | 123.618 | 0.032 | 0.013 |
| Age given drinks other than breastmilk (weeks) | -0.009 | 0.004 | 0.013 | 0.022 |
| **Weight-for-Length z-score** |  |  |  |  |
| Mother age at delivery (yrs) | 0.168 | 0.078 | 0.033 | 0.012 |
| Trimester 2 protein (%TE) | -0.227 | 0.114 | 0.047 | 0.011 |
| Age given drinks other than breastmilk (weeks) | -0.010 | 0.004 | 0.014 | 0.021 |
| 2 year mother weight (kg) | 0.013 | 0.005 | 0.005 | 0.025 |
| 2 year mother BMI (kg/m^2^) | 410.646 | 138.307 | 0.003 | 0.028 |
| **BMI-for-Age z-score** |  |  |  |  |
| Ethnicity | 0.297 | 0.149 | 0.046 | 0.009 |
| Trimester 1 GI | -0.052 | 0.017 | 0.003 | 0.030 |
| Macrosomia (y/n) | -0.377 | 0.136 | 0.006 | 0.020 |
| 2 year mother weight (kg) | 0.013 | 0.005 | 0.018 | 0.017 |
| 2 year mother BMI (kg/m^2^) | 434.032 | 131.928 | 0.001 | 0.035 |
| **Length-for-Age z-score** |  |  |  |  |
| Trimester 2 saturates (%TE) | 0.302 | 0.119 | 0.011 | 0.019 |
| **Mid-Upper Arm Circumference-for-Age z-score** |  |  |  |  |
| Study group (intervention/control) | -0.292 | 0.127 | 0.022 | 0.013 |
| Baseline Moderate activity (no of 20min intervals/week) | 0.097 | 0.045 | 0.032 | 0.020 |
| Mother baseline smoker (y/n) | 0.952 | 0.444 | 0.033 | 0.011 |
| Trimester 2 GI | -0.039 | 0.018 | 0.031 | 0.014 |
| Moderate activity (min/week) | 0.005 | 0.002 | 0.032 | 0.020 |
| Macrosomia (y/n) | -0.283 | 0.129 | 0.029 | 0.011 |
| Duration breastfeeding (weeks) | 0.006 | 0.003 | 0.027 | 0.012 |
| **Waist Circumference:Length Ratio** |  |  |  |  |
| Minutes sitting/weekday | 4.416E-05 | 0.000 | 0.006 | 0.024 |
| Mother height (cm) | 0.000 | 0.000 | 0.009 | 0.017 |
| Achieved 3rd level (y/n) | 0.013 | 0.005 | 0.018 | 0.015 |
| Trimester 1 protein (%TE) | 0.002 | 0.001 | 0.041 | 0.012 |
| Trimester 2 polyunsaturated fat (%TE) | -0.004 | 0.002 | 0.020 | 0.016 |
| Trimester 3 polyunsaturated fat (%TE) | -0.004 | 0.002 | 0.013 | 0.018 |
| Infant gender | 0.013 | 0.005 | 0.012 | 0.016 |
| **Sum of all Skinfold Thicknesses** |  |  |  |  |
| Mother height (cm) | -0.244 | 0.082 | 0.003 | 0.030 |
| Trimester 1 GI | -0.349 | 0.136 | 0.011 | 0.026 |
| Infant gender | 2.436 | 0.999 | 0.015 | 0.019 |
| **Sum of Subscapular and Triceps Skinfold Thicknesses** |  |  |  |  |
| Mother height (cm) | -0.088 | 0.034 | 0.010 | 0.022 |
| Trimester 1 GI | -0.134 | 0.057 | 0.019 | 0.022 |
| **Subscapular:Triceps Skinfold Thickness Ratio** |  |  |  |  |
| Trimester 1 GI | 0.009 | 0.004 | 0.012 | 0.025 |
| Trimester 2 total Fat (%TE) | 0.007 | 0.003 | 0.013 | 0.024 |
| Trimester 2 saturated fat (%TE) | 0.011 | 0.005 | 0.019 | 0.021 |
| Trimester 2 monounsaturated fat (%TE) | 0.016 | 0.006 | 0.012 | 0.024 |
| Infant age at exam (weeks) | 0.007 | 0.002 | 0.000 | 0.058 |

Simple linear regression analysis carried out. Maternal dietary intake derived from food diaries in each trimester of pregnancy. TE total energy, GI glycaemic index, BMI body mass index
