# Supplementary material for: The association between maternal nutrition and lifestyle during pregnancy and 2-year-old offspring adiposity: analysis from the ROLO study
Source: Z Gesundh Wiss. 2016 Jun 9;24(5):427–36. doi: 10.1007/s10389-016-0740-9 (PMC5025498; doi:10.1007/s10389-016-0740-9)
Supplement: Supplementary file 3 — (DOCX 18 kb) [file 10389_2016_740_MOESM3_ESM.docx]

**Online Resource 3** Maternal macronutrient intake in each trimester of pregnancy with comparison of the control and intervention groups

|  |  | Intervention | | Control | | Total | |  |
| --- | --- | --- | --- | --- | --- | --- | --- | --- |
|  | N | mean | SD | mean | SD | Mean | SD | p-value |
| Trimester 1 protein (%TE) | 273 | 17.39 | 3.01 | 16.26 | 2.83 | 16.80 | 2.97 | 0.002 |
| Trimester 2 protein (%TE) | 281 | 18.12 | 3.12 | 16.42 | 2.50 | 17.23 | 2.94 | <0.001 |
| Trimester 3protein (%TE) | 281 | 17.73 | 3.13 | 16.39 | 2.77 | 17.04 | 3.02 | <0.001 |
| Trimester 1 carbohydrate (%TE) | 273 | 50.59 | 6.95 | 50.75 | 6.62 | 50.67 | 6.77 | 0.849 |
| Trimester 2 carbohydrate (%TE) | 281 | 49.25 | 5.86 | 50.24 | 6.13 | 49.76 | 6.01 | 0.168 |
| Trimester 3 carbohydrate (%TE) | 281 | 48.89 | 5.32 | 50.61 | 6.26 | 49.78 | 5.88 | 0.014 |
| Trimester 1 total fat (%TE) | 273 | 35.04 | 5.84 | 35.99 | 5.70 | 35.54 | 5.78 | 0.174 |
| Trimester 2 total fat (%TE) | 281 | 35.54 | 5.07 | 36.30 | 5.46 | 35.94 | 5.28 | 0.231 |
| Trimester 3 total fat (%TE) | 281 | 36.23 | 4.95 | 35.86 | 5.33 | 36.04 | 5.14 | 0.543 |
| Trimester 1 saturated fat (%TE) | 273 | 13.39 | 3.08 | 13.83 | 2.88 | 13.62 | 2.98 | 0.223 |
| Trimester 2 saturated fat (%TE) | 281 | 13.27 | 3.03 | 13.84 | 2.88 | 13.57 | 2.96 | 0.106 |
| Trimester 3 saturated fat (%TE) | 281 | 13.90 | 2.76 | 13.81 | 3.06 | 13.86 | 2.91 | 0.801 |
| Trimester 1 polyunsaturated fat (%TE) | 273 | 5.91 | 2.08 | 5.79 | 2.27 | 5.85 | 2.18 | 0.649 |
| Trimester 2 polyunsaturated fat (%TE) | 281 | 5.92 | 1.77 | 5.89 | 1.80 | 5.91 | 1.78 | 0.872 |
| Trimester 3 polyunsaturated fat (%TE) | 281 | 5.87 | 1.80 | 5.68 | 1.72 | 5.77 | 1.76 | 0.344 |
| Trimester 1 monounsaturated fat (%TE) | 273 | 10.99 | 2.45 | 11.40 | 2.50 | 11.20 | 2.48 | 0.182 |
| Trimester 2 monounsaturated fat (%TE) | 281 | 11.16 | 2.39 | 11.46 | 2.24 | 11.31 | 2.31 | 0.278 |
| Trimester 3 monounsaturated fat (%TE) | 281 | 11.27 | 2.22 | 11.22 | 2.18 | 11.24 | 2.19 | 0.836 |
| Trimester 1 energy (MJ) | 273 | 7.51 | 1.71 | 7.96 | 1.77 | 7.75 | 1.76 | 0.033 |
| Trimester 2 energy (MJ) | 281 | 7.42 | 1.85 | 8.29 | 1.69 | 7.87 | 1.82 | <0.001 |
| Trimester 3 energy (MJ) | 281 | 7.65 | 1.69 | 8.39 | 1.84 | 8.03 | 1.81 | 0.001 |
| Trimester 1 glycaemic index | 273 | 57.26 | 4.38 | 57.74 | 3.48 | 57.51 | 3.94 | 0.324 |
| Trimester 1 glycaemic load | 273 | 131.26 | 33.59 | 140.00 | 37.28 | 135.83 | 35.77 | 0.046 |
| Trimester 2 glycaemic index | 281 | 56.26 | 3.94 | 57.59 | 3.33 | 56.96 | 3.69 | 0.003 |
| Trimester 2 glycaemic load | 281 | 121.17 | 31.60 | 143.54 | 31.69 | 132.86 | 33.51 | <0.001 |
| Trimester 3 glycaemic index | 281 | 55.74 | 3.64 | 57.56 | 3.81 | 56.69 | 3.83 | <0.001 |
| Trimester 3 glycaemic load | 281 | 124.08 | 30.39 | 145.62 | 37.35 | 135.33 | 35.80 | <0.001 |

Comparison of control and intervention groups carried out using independent sample tests. TE total energy
